# Targeting *KRAS* in Cancer: Promising Therapeutic Strategies

**DOI:** 10.3390/cancers13061204

**Published:** 2021-03-10

**Authors:** Lisa Maria Mustachio, Anca Chelariu-Raicu, Lorant Szekvolgyi, Jason Roszik

**Affiliations:** 1 Department of Epigenetics and Molecular Carcinogenesis, University of Texas MD Anderson Cancer Center, Houston, TX 77030, USA; 2 Center for Cancer Epigenetics, University of Texas MD Anderson Cancer Center, Houston, TX 77030, USA; 3 Department of Obstetrics and Gynecology, University Hospital, Ludwig Maximilian University of Munich, 80539 Munich, Germany; AChelariu@mdanderson.org; 4 Genome Architecture and Recombination Research Group, Department of Biochemistry and Molecular Biology, MTA-DE Momentum, Faculty of Medicine, University of Debrecen, 4002 Debrecen, Hungary; lorantsz@med.unideb.hu; 5 Department of Genomic Medicine, Division of Cancer Medicine, University of Texas MD Anderson Cancer Center, Houston, TX 77030, USA; 6 Department of Melanoma Medical Oncology, Division of Cancer Medicine, University of Texas MD Anderson Cancer Center, Houston, TX 77030, USA

**Keywords:** cancer, EGFR, *KRAS*, MAPK, mutations, targeted-therapy

## Abstract

**Simple Summary:**

Since the Kirsten rat sarcoma viral oncogene homolog (*KRAS*) is mutated in about 25% of all human cancers and is at the center of pathways involved in tumorigenesis, it is necessary to compile and highlight the novel therapeutic strategies behind targeting this oncoprotein in cancer. Over the years, many have studied various methods to directly target *KRAS* with no success. Fortunately, there has been more success in targeting other proteins along the RAS pathway to yield a therapeutic response. However, some recent findings show promising results indicating that we are one step closer to developing an effective inhibitor that directly targets *KRAS*. The review presented here summarizes these recent findings and emphasizes the need to continue the search for the most optimal *KRAS* inhibitor that can be used to treat and potentially even cure certain tumor types.

**Abstract:**

The Kirsten rat sarcoma viral oncogene homolog (*KRAS*) is mutated in approximately 25% of all human cancers and is known to be a major player promoting and maintaining tumorigenesis through the RAS/MAPK pathway. Over the years, a large number of studies have identified strategies at different regulatory levels to tackle this ‘difficult-to-target’ oncoprotein. Yet, the most ideal strategy to overcome *KRAS* and its downstream effects has yet to be uncovered. This review summarizes the role of *KRAS* activating mutations in multiple cancer types as well as the key findings for potential strategies inhibiting its oncogenic behavior. A comprehensive analysis of the different pathways and mechanisms associated with *KRAS* activity in tumors will ultimately pave the way for promising future work that will identify optimum therapeutic strategies.

## 1. Introduction

Mutations in Kirsten rat sarcoma viral oncogene homolog (*KRAS*) are one of the most common oncogenic events in endodermal carcinomas [1]. The *KRAS* gene can simultaneously harbor multiple mutations that can potentiate tumor-promoting activity [2]. In fact, alterations in *KRAS* have been identified in 25% of all cancers, where some cancers like pancreatic cancer contain extremely high mutation rates (90%), while others, such as prostate cancer, show lower mutation rates (7%) [1,3]. Not only do *KRAS* mutations promote and maintain tumorigenesis, they also increase the chance of resistance and poor prognosis, ultimately contributing to over one million deaths annually [1,3]. Even though mutations in other RAS isoforms, including the neuroblastoma RAS viral (v-ras) oncogene homolog (*NRAS*) and Harvey rat sarcoma viral oncogene homolog (*HRAS*) are prevalent in many cancer types, mutations in *KRAS* account for 85% of all RAS isoform mutations [1,3].

Scientists have been aiming to target *KRAS* for decades but have experienced difficulty in identifying strategies to target the smooth surface of the protein [4]. Activating mutations in *KRAS* increase the ability for GTP-loading and the GTP-binding pocket holds onto its substrate very tightly, making it difficult to displace [1,4]. Thus, constitutive activation of downstream signaling of the mitogen-activated protein kinase (MAPK) pathway occurs and regulates proteins involved in cell proliferation, development, inflammation, differentiation and apoptosis to promote cancer formation [1,3]. As a result, indirect strategies targeting downstream signaling of *KRAS* were attractive options but have still not resulted in desired outcomes. The epidermal growth factor receptor (EGFR) upstream of *KRAS* is also frequently mutated in various cancers and has also been sought as a therapeutic strategy to tackle *KRAS* mutant cancers [3]. Despite the seemingly many options to target *KRAS*, there have been many failed attempts in the past.

**Figure 1:**
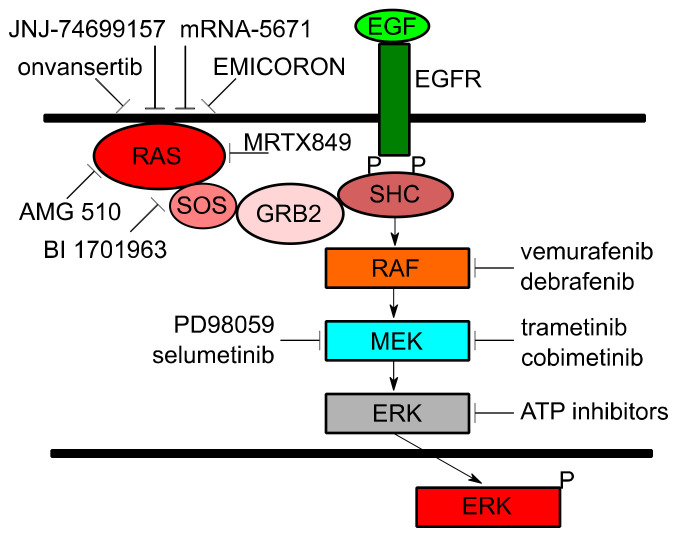
The role of *KRAS* and current agents in clinical or preclinical development.

However, with more studies various new strategies have been identified to target *KRAS* for cancer therapy [4]. New associations between *KRAS* and certain cancer phenotypes as well as the development of new agents are providing more hope in the battle against this difficult-to-target oncoprotein. This review will provide a summary of *KRAS* and its role in various tumors and provide a compilation of selected new insights and agents currently in the preclinical or clinical stages. (Figure 1) to treat cancers harboring *KRAS* mutations.

## 2. Blood Cancers

*RAS* mutations are identified in ~15% of acute myeloid leukemia (AML), 11% of adult T lineage acute lymphoblastic leukemia (T-ALL) and 30–40% of multiple myeloma (MM) patients [5]. Approximately 5% of AML cases harbor *KRAS* mutations and these mutations are thought to be early or initiating events in disease formation as well as acquired mutations during disease progression [5,6]. Overall survival and complete remission rate in AML patients are negatively influenced by the presence of *KRAS* mutations and *KRAS* was identified as a predictor of prognosis in these patients [7]. Similarly, the presence of *KRAS* mutations as well as mutations in *NRAS* were found to be associated with a shorter treatment-free survival in trisomy 12 chronic lymphocytic leukemia cases [8]. In pediatric B-cell precursor acute lymphoblastic leukemia (BCP-ALL), *KRAS* is mutated in approximately 44% of cases and is linked to relapse as well as chemotherapy resistance [9]. Interestingly, whole-genome sequencing revealed a 67% mutation rate in genes associated with the RAS signaling pathway [5]. In MM patients, *KRAS* mutations comprise 23% of cases and predict shorter overall survival as well as shorter progression-free survival [5].

Despite the high *KRAS* mutation rate in various leukemias and myeloma cases, the development of therapeutics specifically targeting *KRAS* has been a challenge. One study showed that high doses of cytarabine decrease the chance of relapse in *RAS*-mutated AML cases [10,11]. However, an attractive strategy to overcome *KRAS* is to target downstream effectors or other proteins in the RAS pathway. Homozygous mutant *KRAS* AML is less sensitive to mitogen-activated protein kinase kinase (MEK) inhibitors than cells containing a wild-type *KRAS* allele [12]. Even though attempts have been made to target MEK, mechanistic target of rapamycin (mTOR), protein kinase B (AKT), phosphatidylinositol 3-kinase (PI3K) and rapidly accelerated fibrosarcoma (RAF) to inhibit RAS signaling, no optimal strategies were identified since inhibition of one of these proteins results in the stimulation of compensatory mechanisms [13]. It is also important to mention that the RAS/RAF/MEK/extracellular signal-regulated kinase (ERK) pathway is activated in response to the administration of other drugs commonly used to treat leukemia [14]. Thus, targeting *KRAS* and its pathway in cancers of the blood has been challenging. Some preclinical studies have shown great promise in developing new methods to target the RAS pathway. One study unveiled that a novel competitive ATP inhibitor targeting ERK1 and ERK2 may have clinical benefit in *KRAS* mutant AML cases [14]. Inhibition of the *KRAS* activator SOS Ras/Rac guanine nucleotide exchange factor 1 (SOS1) is an effective approach when treating cancers driven by *KRAS* [15]. In addition, inhibiting SOS1 increases the sensitivity of *KRAS* mutant cancers to MEK inhibition. Since some AML cell lines harbor *SOS1* mutations and are dependent on SOS1 for survival, a combination of SOS1 and MEK inhibitors may be an attractive strategy for the treatment of blood cancers containing *KRAS* mutations [16].

Interestingly, a new study revealed that the activation of *KRAS* wild-type alone is sufficient to induce oncogenic *KRAS* properties in cases of Down Syndrome (DS)-ALL [17]. Approximately 60% of DS-ALL cases harbor cytokine receptor-like factor 2 (CRLF2) rearrangements, 32% of cases harbor *JAK2* mutations and 36% harbor mutations in the *RAS*-*MAPK* pathway. Both *JAK2* and *RAS* pathway mutations are mutually exclusive. However, high CRLF2 levels and increased activation of JAK2 sufficiently activate wild-type *KRAS* in 80% of DL-ALL cases, suggesting that therapeutics inhibiting overstimulated *KRAS* are needed [17].

## 3. Breast Cancer

For quite some time, the role of *KRAS* in breast cancer has not been a point of focus since *KRAS* mutations are infrequent in this cancer type. However, out of all RAS proteins, *KRAS* is the most frequently mutated RAS protein in breast cancer and is associated with poor prognosis as well as increased rate of metastasis [18]. More recent findings show that *KRAS* plays a role in promoting the activation of certain pathways involved in oncogenic behaviors contributing to breast tumorigenesis. For example, *KRAS* and PI3K have been found to cooperate in the production of de novo lipid biogenesis and RAS oncogenesis is exaggerated when the cyclin-dependent kinase inhibitor (p21WAF1/CIP1) is depleted in breast cancer [18,19,20]. Despite not frequently mutated in breast cancer, RAS proteins are known to be upregulated in breast tumors when compared to adjacent normal breast tissues and are correlated with p185/human epidermal growth factor receptor 2 (HER2) expression [21,22]. Both HER2 as well as EGFR are frequently overexpressed in breast cancer, amplify the RAS signaling pathway and are therapeutically targeted to treat this cancer type [18]. In another example, miRNA-382-5p enhances the progression of breast cancer through regulation of the RAS-like estrogen regulated growth inhibitor (RERG)/RAS/ERK axis [23]. Thus, even in the absence of genetic mutations, *KRAS* overexpression and signaling still contributes to breast oncogenesis. In addition to upstream effectors influencing RAS activity, certain regulators of *KRAS*, such as R-RasGTPases, mediate the interaction between estrogen and insulin signaling in breast cancer cells [24]. In addition, RAS protein activator like 2 (RASAL2), a RasGAP gene, promotes the progression of triple-negative breast cancer through the activation of RAS-related C3 botulinum toxin substrate (RAC1) [25]. Specifically, many studies have linked RAS activity to the promotion of triple-negative breast cancer. For one, *KRAS* variants regulate the development of triple-negative breast cancer [26]. The RAS-MAPK pathway allows for immune-evasion in triple-negative breast cancer [27] and ERK1/2 phosphorylation levels are significantly increased in metastasized tumors stemming from a triple-negative breast primary tumor [28].

Currently, inhibitors have been produced that target downstream effector pathways of *KRAS*, including MEK inhibitors that are used to treat triple-negative breast cancer [29]. MEK inhibitors have shown some promising results in the treatment of other cancers. However, in breast cancer, MEK inhibitors do not improve overall survival [30] One study revealed that the MEK inhibitor PD98059 inhibited breast cancer cell proliferation but contributed to increased migration ability, explained by increased levels of β-catenin levels in the nucleus [30]. One certain fact is that the role of RAS in breast cancer cannot be ignored since it also has been associated with increased levels of resistance to cisplatin [31] and the tyrosine kinase inhibitor lapatinib [32] as well as influencing the response to tamoxifen [33].

## 4. Colorectal Cancer

The three *RAS* genes are mutated in 45% of all colorectal cancer cases, where *KRAS* is the most frequently mutated RAS isoform. Similarly, EGFR is overexpressed in 60–80% of colorectal cancers and specific antibody therapies, including cetuximab and panitumumab, have been used against EGFR and to target its downstream RAS/RAF/MEK/ERK signaling pathway [34,35]. However, with time, colorectal cancer patients experience resistance to EGFR inhibitors due to ability of RAS to activate the same downstream signaling pathway as EGFR that results through acquired resistance [36,37]. This suggests that the issue of *KRAS* activation needs to be tackled before EGFR inhibitors will be fully effective in more colorectal cancer cases. In light of these findings, the American Society of Clinical Oncology (ASCO) recommends testing both the EGFR and *KRAS* mutation status in patients with metastatic colorectal cancer who are candidates for anti-EGFR antibody therapy [38]. There have been various strategies aiming to directly target *KRAS* by altering its interaction with the cellular plasma membrane. However, farnesyl transferase inhibitors that made it to clinical trials appeared to be ineffective in treating cancer, including colorectal cancer cases [39]. There are some promising in vitro results showing that inhibitors against prenyl-binding protein PDEδ, which prevents *KRAS* from localizing to the plasma membrane, inhibit the growth of *KRAS* mutant tumor cell lines [40,41].

Numerous attempts are still being made to directly target *KRAS*. One method is inhibiting the interaction between RAF and GTP-bound *KRAS*, which has shown strong anti-tumor activity in *KRAS G12V* mutated colorectal cancer xenografts [42]. The use of synthetic alkylating agents recognizing and alkylating adenine residues of mutant *KRAS* have been developed and showed to inhibit the proliferation of *KRAS* mutant versus *KRAS* wild-type colorectal cancer cell lines [43]. Since the direct targeting of *KRAS* has been difficult in all cancers, inhibitors focusing on targeting RAF/MEK/ERK have been generated. Pan-inhibitors that inhibit b-RAF proto-oncogene (BRAF) and c-RAF proto-oncogene (CRAF) as well as not activating the MAPK pathway in cancers harboring *KRAS* mutations are being generated and show in vitro promising results [44]. *KRAS* mutant colorectal cancers have shown to be sensitive to a more recent G4 ligand known as EMICORON [41]. It was also shown that EMICORON in combination with chemotherapy improved anti-tumor efficacy as well as decreased both mRNA and protein expression levels of *KRAS* in colorectal cancer PDXs [34]. Lastly, another strategy to focus on regulating miRNAs associated with *KRAS* and its functions. For example, reduced levels of miR-143 increase cancer phenotypes of colorectal cancer cells [45].

One of the most promising therapies for the treatment of *KRAS* mutant colorectal cancers is onvansertib, a selective adenosine triphosphate competitive inhibitor of serine/threonine polo-like-kinase 1 (PLK1). It is currently being tested in a Phase 1b/2 study along with the chemotherapeutic FOLFIRI and Avastin, which binds to vascular endothelial growth factor (VEGF) as a second-line treatment for metastatic colorectal cancer patients harboring *KRAS* mutations (Clinicaltrials.gov Identifier: NCT03829410 (accessed on 24 February 2021)). FOLFIRI is also being tested with MEK162 to evaluate the response rate, clinical benefit and safety parameters in advanced *KRAS* positive metastatic colorectal cancers (Clinicaltrials.gov Identifier: NCT02613650 (accessed on 24 February 2021)). Similarly, FOLFIRI is also being tested in combination with the virus based investigative therapy REOLYSIN and bevacizumab in FOLFIRI naïve patients with *KRAS* mutant metastatic colorectal cancer (Clinicaltrials.gov Identifier: NCT01274624 (accessed on 24 February 2021)). Patients enrolled in this study have shown a 100% clinical benefit. This study is expected to be completed in May 2022. In addition, the direct *KRAS G12C* inhibitor AMG 510, which will be described more in the lung and pancreatic cancer sections, has also showed great promise in reducing tumor size in colorectal cancer patients [46]. Lastly, the combination of the kinase inhibitor sorafenib and chemotherapeutic irinotecan (NEXIRI) has shown promising results as a second or later-line treatment for metastatic colorectal cancer patients with *KRAS* mutations as well as a treatment option for patients refractory to standard combined chemotherapies [47,48].

## 5. Gynecological Cancers

Gynecological malignancies account for 15–20% of all malignancies in women worldwide [49]. As in most cancers, genomic profiling of gynecological cancers has revealed new targets for tumor-specific treatment and the demand for individualized cancer therapy has increased in recent years. Spaans et al., investigated somatic mutations in gynecological cancers and found that *PIK3A*, *PTEN* and *KRAS* were the most frequently occurring mutations, with rates being 22, 18 and 12%, respectively [50]. Histologic types of ovarian cancer have been associated with different genetic alternations and both *KRAS* and *BRAF* mutations have been found in low-grade serous ovarian cancer (LGSOC), 16 to 44% *KRAS* mutation and 2 to 20% *BRAF* mutation [51]. In studies investigating cervical cancer samples, *KRAS* mutations were identified as the second common oncogenic mutation following PIK3CA, although a prevalence of 2% makes it a rare event [52]. Nevertheless, *KRAS* mutations seem to be directly associated with type I estrogen-related endometrial cancer and its frequency is estimated to be 10–30% [53].

While genomic studies have shown that the *KRAS* pathway plays a key role in several gynecological cancers, the exact mechanism of tumorigenesis involving *KRAS* mutations has been mostly investigated in studies focusing on endometrial cancer and low-grade ovarian cancer. Mechanisms such as the upregulation of endometrial cell estrogen receptors through the *KRAS* pathway and hypermethylation-related changes in the *KRAS* promoter are well-demonstrated events in endometrial carcinogenesis [54,55]. *KRAS* mutations have been identified in early stage endometrial hyperplasia specimens [56] and due to their biological function to support tumor proliferation, by assessing the presence of this mutation may offer an opportunity to predict tumor invasiveness [57]. Similarly, *KRAS* mutation that range from 17 to 39.5% in ovarian serous borderline tumors, may represent early events in the tumorigenesis of the LGSOC [58]. In contrast, *BRAF* mutations with a frequency until 33% in ovarian serous borderline tumors, appear to have a less important role in the progression of low-grade serous ovarian cancer [58]. Taken together, these findings suggest that targeting *KRAS* pathway represents an exciting and promising new direction of therapy for gynecological cancers, including endometrial and LGSOC.

Several clinical trials from phase I to phase III targeting the *KRAS* pathway have been developed. Most treatment strategies act at the post-transcriptional level and target downstream effectors of *KRAS*, such as MEK1/2, a critical kinase in the mitogen-activated protein kinase signal transduction pathway. As observed in phase II trial investigating selumetinib in the treatment of recurrent or persistent endometrial cancer, single agent therapy in a non-selected population was unlikely to make a measurable difference in outcome [59]. More promising results were shown by a phase II trial investigating the same drug, selumetinib, in LGSOC [60]. The treatment was active in 15% of patients who showed a partial response. Additionally, 65% of patients demonstrated clinical benefit by showing stable disease. On the basis of this positive trial, two large randomized clinical trials of MEK inhibitors in recurrent LGSOC were designed, evaluating the efficacy of two different MEK compared with standard of care treatments, including pegylated liposomal doxorubicin or weekly paclitaxel, topotecan, letrozole or tamoxifen. Only one of the trials met its primary endpoint, showing a clinical advantage of trametinib (HR, 0.48; *p* < 0.001) (Clinicaltrials.gov Identifier: NCT02101788 (accessed on 24 February 2021)). A correlation between *KRAS*/BRAF mutations and clinical outcome analysis is ongoing. This mutational analysis will be very important, given the fact that integrated biomarker-based trials represent the most likely strategy to fully realize the potential therapeutic efficacy of targeted therapy in gynecological cancer with less frequency of alterations in the *KRAS* pathway.

## 6. Lung Cancer

Lung cancers account for the highest cancer incidence rates behind gender specific cancers, such as prostate or breast cancers, as well as contribute to the greatest number of cancer-related deaths. Non-small cell lung cancer (NSCLC) is the most common type of lung cancer accounting for about 85% of all lung cancer cases and *KRAS* mutations are the most frequent alteration in these cancers [61]. More specifically, mutations in *KRAS* are present in up to 40% of lung adenocarcinomas [61]. Multiple oncogenic events have been of focus in NSCLC, including proteins upstream of *KRAS*, such as EGFR, as well as downstream effector proteins, such as BRAF [62]. Inhibitors and therapeutic strategies against these proteins, as well as others involved in various pathways have been developed and are showing promising results for the future [62]. However, as with most therapies used for NSCLC, the risk of resistance is high due to increased chances of compensatory mechanisms that evolve with time. Thus, identifying strategies to inhibit key players, such as *KRAS*, in this cancer type are necessary for novel alternatives.

Over the last few years, breakthroughs have been made in identifying novel strategies to target *KRAS* and lung cancer has been a primary model of study to determine the effectiveness of these new agents. The compound AMG 510 (sotorasib), developed by Amgen, uses an irreversible occupation of the His95 groove near the cysteine pocket to inactivate *KRAS* [46]. This compound is an improved version derived from the covalent compound ARS-1620 [63]. AMG 510 significantly inhibits *KRAS G12C* and MAPK signaling in lung cancer cell lines but does not seem to influence wild-type *KRAS*. Initial results from a phase I/phase II clinical trial consisting of 533 patients with advanced NSCLC harboring *KRAS G12C* mutations as well as other solid tumors with *KRAS G12C* mutations has revealed that AMG 510 exerts a partial response in over 50% and a full response in 46% of the patients studied. This is an ongoing trial with a completion date set for spring of 2024 (Clinicaltrials.gov Identifier: NCT03600883 (accessed on 24 February 2021)). Overall, AMG 510 shows encouraging anticancer activity with minimal toxicity in patients that were previously treated for advanced solid tumors harboring *KRAS G12C* mutations [10]. However, *KRAS G12C* signaling has been shown to be briefly suppressed by inhibitors and after a short time, re-accumulation of active *KRAS* and ERK signaling occurs. New *KRAS G12C* can be produced is remains active and insensitive to drugs, resulting in a population of cells that do not respond to treatment [64]. This reactivation occurs through compensatory activation of receptor tyrosine kinases and SOS1/2 [65]. Future strategies must consider blocking new *KRAS* through these compensatory mechanisms and some new strategies are discussed below.

Other inhibitors against *KRAS* have been identified over the last couple of years that show promising results in lung cancer. The pyrazolopryimidine-based inhibitor Compound 11 has a high affinity to the allosteric p1 pocket of *KRAS* [66] and has shown disruption of MAPK signaling. The novel Mirati Therapeutics (San Diego, CA, USA) compound MRTX849 is currently in clinical trials as a *KRAS G12C* inhibitor and contains a similar structure to AMG 510. MRTX849 was shown to be an effective monotherapy in lung cancer cells lines and *KRAS* mutant lines showed to be more sensitive than wild-type cell lines. In an ongoing trial, patients with advanced lung cancer have shown a partial response when treated with MRTX849 and this trial is to be completed by the end of 2021 (Clinicaltrials.gov Identifier: NCT03785249 (accessed on 24 February 2021)). The *KRAS G12C* inhibitor known as JNJ-74699157 is currently being tested in a phase I clinical trial to determine the tolerated dose in patients with advanced solid tumors, including lung cancer (Clinicaltrials.gov Identifier: NCT04006301 (accessed on 24 February 2021)).

Another inhibitor currently being tested in patients and that will be completed in 2023 is the *KRAS*-SOS1 inhibitor BI 1701963 that is being analyzed as a monotherapy as well as with the MEK inhibitor trametinib to evaluate the maximum tolerated dose, safety and efficacy (Clinicaltrials.gov Identifier: NCT04111458 (accessed on 24 February 2021)). TVB-2640, a selective and reversible inhibitor of fatty acid synthase (FASN) that exerts effects on *KRAS* mutant versus *KRAS* wild-type NSCLCs, is currently being tested in a phase 2 study that includes NSCLC patients harboring *KRAS* mutations since *KRAS* mutant cases are associated with lipogenic features (Clinicaltrials.gov Identifier: NCT03808558 (accessed on 24 February 2021)). Lastly, the mRNA-5671 cancer vaccine for *KRAS G12C*, *G12D*, *G13D* and *G12V* is currently being tested in a phase I study as a monotherapy as well as with the PD1 inhibitor pembrolizumab in advanced or metastatic *KRAS* mutant NSCLC, colorectal and pancreatic cancer patients with an estimated study completion date being end of 2026 (Clinicaltrials.gov Identifier: NCT03948763 (accessed on 24 February 2021)) [4].

The fibroblast growth factor 1 (FGFR-1) inhibitor ponatinib is currently being tested in combination with trametinib in *KRAS* mutant NSCLC patients since it is known that this combination induces cell death in *KRAS* mutant positive tumors in vivo [67] (Clinicaltrials.gov Identifier: NCT03704688 (accessed on 24 February 2021)). In addition, trametinib is being tested in combination with the chemotherapeutic docetaxel in patients with recurrent or Stage IV *KRAS* mutant NSCLC (Clinicaltrials.gov Identifier: NCT02642042 (accessed on 24 February 2021)). In addition, there are various potential *KRAS* inhibitors in the preclinical stage that are currently being studies and may result in some strong leads in the near future [4]. Various other alternatives targeting *KRAS* indirectly through protein degradation pathways are also attractive strategies for the future [68,69].

## 7. Pancreatic Cancer

*KRAS* mutations are found in 90% of pancreatic ductal adenocarcinomas (PDACs) and are known to be an initiating event for this aggressive cancer type [46]. In the past, various approaches were studied to indirectly target *KRAS* in PDAC. Some of these techniques included targeting small GTPase effectors, such as the RACGEF-RAC1 and RACGEF-RAL pathways or focusing on targeting the mTOR or RAF/MEK/ERK signaling pathways [46]. In fact, various BRAF and MEK1/2 inhibitors are under clinical investigation and a handful have been improved for use in *BRAF*-mutant melanoma [46]. However, inhibitors of *KRAS* downstream effectors have not shown to be sufficient for the treatment of PDAC patients. A randomized phase II trial comparing the MEK inhibitor selumetinib and the AKT inhibitor MK-2206 to the chemotherapeutic FOLFIRINOX for metastatic PDAC patients showed inferior efficacy and increased adverse reactions [70]. A phase II study investigating REOLYSIN in combination with gemcitabine for chemotherapy naïve patients with advanced pancreatic adenocarcinoma showed a favorable response and will be tested in the future with anti-PDL1 therapies [71]. An ongoing trial being conducted by M.D. Anderson Cancer Center is testing the autophagy inhibitor hydroxychloroquine along with the MEK inhibitor binimetinib for the treatment of patients with *KRAS* mutant metastatic pancreatic cancer (Clinicaltrials.gov Identifier: NCT04132505 (accessed on 24 February 2021)). Selumetinib sulfate is also being tested in patients with locally advanced or metastatic pancreatic cancer harboring *KRAS G12R* mutations (Clinicaltrials.gov Identifier: NCT03040986 (accessed on 24 February 2021)).

Evaluation of AMG 510 for the treatment of PDAC revealed significant inhibition of *KRAS G12C* and MAPK signaling in PDAC cell lines, with no effect on wild-type *KRAS*. As mentioned, AMG 510 is currently being tested in a larger trial and is showing to have promising results, but its effects on PDAC may be limited since the *KRAS G12C* mutation only occurs in 2% of PDAC cases [46]. Similarly, MRTX849 has shown promising effects in PDAC cell lines and it will be interesting to see how it influences the treatment of PDAC patients [72]. Lastly, some studies have shown a synergistic response between *KRAS* and autophagy inhibitors in PDAC models [73]. These may provide novel strategies for the treatment of PDAC moving forward.

## 8. Prostate Cancer

Prostate cancer is the most common tumor and the second cause of cancer-related deaths in males. Mutations in *KRAS* account for 7% of prostate cancer cases and have been linked to effector proteins such as RAF, PI3K and GDP/GTP Ral exchange factor, contributing to downstream signaling responses. Mutant *KRAS* is known to be a transformative factor in prostate cancer and found to promote cancer stemness and bone metastasis [74,75,76]. In addition, *KRAS* rearrangements have been shown to promote the metastatic progression of prostate cancer [77].

Similar to what has been described for blood cancers, there are no therapies directly targeting *KRAS* in prostate cancer, but other proteins upstream and downstream of *KRAS* have attracted interest as potential targets. Targeting EGFR with inhibitors such as gefitinib, lapatinib and erlotinib has shown limited effectiveness in prostate cancer and more recent studies reveal that the extracellular release of EGFR through exosomes may be the culprit [78,79]. A recent study revealed that hyperactive ERK1 is present in a large number of refractory prostate cancer cases and these cases also have frequent amplifications in the MAPK pathway, suggesting that MEK/ERK can be pharmacologically targeted to treat refractory metastatic prostate cancer [80]. Future clinical trials will confirm whether targeting MEK/ERK in *KRAS* mutant cancers will be effective in the treatment of prostate cancer.

## 9. Skin Cancer

In melanoma, mutations in *KRAS* are rare, only accounting for 1.7% of cases [81]. However, mutations in *BRAF* are prominent and inhibition of BRAF is a common, standard therapy for the treatment of *BRAF*-mutant melanoma. Unfortunately, only 50% of patients containing *BRAF* mutations respond to therapy and there are only slight improvements in overall survival. Recent work has shown that inhibition of wild-type *KRAS* in *BRAF*-mutant melanoma can be a potential strategy since inhibition of *KRAS* works synergistically with BRAF inhibition to reduce the proliferation and induce apoptosis independent of *BRAF* mutation status [82]. Similar to melanoma, *KRAS* is found to be mutated in skin squamous cell carcinoma but at a low rate. In fact, many mouse models of cutaneous squamous cell carcinoma take advantage of inducible mutations in *KRAS* along with mutations of specific tumor suppressor genes [83]. There has been a great deal of progress in targeting downstream effector proteins of *KRAS* for the treatment of melanoma. For example, there are two BRAF inhibitors, including vemurafenib and dabrafenib, as well as the two MEK1/2 inhibitors trametinib and cobimetinib, that have been approved for *BRAF*-mutant melanoma [46]. This is one example of a success story of targeting downstream effectors of *KRAS* for cancer therapy.

## 10. Conclusions and Future Directions

Dysregulation of the MAPK pathway plays a central role in a number of malignancies and therapeutic inhibition of oncogenic RAS is of great clinical importance. There has been a significant progress made in this area resulting in successful targeting of *KRAS* or downstream effectors in several cancer types, with multiple agents in clinical trials (Table 1). The trials listed in Table 1 are only a handful of selected studies chosen to be highlighted in this review. It is important to note that over 200 results exist when searching for clinical trials related to *KRAS* on Clinicaltrials.gov (accessed on 24 February 2021). A good number of these trials are recruiting and active as well are testing combinations between chemotherapeutics and other drugs that have been shown to attenuate *KRAS* mutant phenotypes in vitro. However, our understanding how *KRAS* inhibitory ligands selectively target protein conformation and how they interfere with the MAPK pathway is most likely still incomplete. In addition, we need to fully understand why acquired *KRAS* mutations are more prominent in certain cancer types. For example, *KRAS* mutations in lung cancer may be more frequent due to exposure to carcinogens, such as tobacco smoke. Overall, the close interaction between preclinical experimental approaches and theoretical approaches, including molecular dynamic simulation, will continue to play a key role in the treatment and the ultimate eradication of *KRAS* mutant tumors.

## Figures and Tables

**Table 1:** Select recent clinical trials targeting *KRAS* and downstream signaling pathways.

Trial ID	Trial Title	Target(s)	Cancer(s)	Mechanism
NCT01274624	Study of REOLYSIN in Combination With FOLFIRI and Bevacizumab in FOLFIRI Naïve Patients With *KRAS* Mutant Metastatic Colorectal Cancer	Activated RAS	CRC	Stimulates lysis of tumor cells and induces antitumor immunity
NCT03808558	Phase 2 Study of TVB-2640 in *KRAS* Non-Small Cell Lung Carcinomas	FASN	NSCLC	Disrupts tumor lipid rafts and RAS localization
NCT03600883	A Phase 1/2, Study Evaluating the Safety, Tolerability, PK, and Efficacy of AMG 510 in Subjects With Solid Tumors With a Specific *KRAS* Mutation (CodeBreak 100)	*KRAS G12C*	NSCLC, solid tumors	Binds to cysteine residue in *KRAS G12C* mutations holding protein in inactive form and prevents downstream signaling
NCT03785249	Phase 1/2 Study of MRTX849 in Patients With Cancer Having a *KRAS G12C* Mutation KRYSTAL-1	*KRAS G12C*	NSCLC, CRC, other solid tumors	Modifies mutant cysteine 12 in a GDP-bound state and inhibits *KRAS* dependent signaling
NCT04006301	First-in-Human Study of JNJ-74699157 in Participants With Tumors Harboring the *KRAS G12C* Mutation	*KRAS G12C*	NSCLC, solid tumors	Binds to *KRAS G23C* and holds the protein in inactive form, preventing downstream signaling
NCT03948763	A Study of mRNA-5671/V941 as Monotherapy and in Combination With Pembrolizumab (V941-001)	*KRAS G12D*, *G12V*, *G13D*, and *G12C*	NSCLC, CRC, pancreatic	Induces T-cell dependent immune responses to destroy tumors presenting *KRAS* mutations
NCT04132505	Binimetinib and Hydroxychloroquine in Treating Patients With *KRAS* Mutant Metastatic Pancreatic Cancer	MEK, Autophagy	pancreatic	Binimetinib: Noncompetitive ATP inhibitor preventing MEK signaling. Hydroxychloroquine: Inhibits autophagy to avoid metabolism of Binimetinib
NCT03040986	Selumetinib Sulfate in Treating Patients With Locally Advanced or Metastatic Pancreatic Cancer With *KRAS G12R* Mutations	MEK	pancreatic	Potent and selective inhibitor against MEK1/2 and *KRAS* dependent signaling
NCT02642042	Trametinib and Docetaxel in Treating Patients With Recurrent or Stage IV *KRAS* Mutation Positive Non-small Cell Lung Cancer	MEK	NSCLC	Trametinib: Inhibits MEK signaling downstream of *KRAS*. Docetaxel: Chemotherapeutic
NCT02101788	Trametinib in Treating Patients With Recurrent or Progressive Low-Grade Ovarian Cancer or Peritoneal Cavity Cancer	MEK	ovarian, peritoneal	Inhibits MEK signaling downstream of *KRAS*
NCT02613650	A Trial of mFOLFIRI With MEK162 in Patients With Advanced RAS (HRAS, NRAS, or *KRAS*) Positive Metastatic Colorectal Cancers	MEK	CRC	MEK162: Uncompetitive ATP inhibitor suppresses the activity of MEK1/2 and KRAS downstream signaling. mFOLFIRI: Chemotherapeutic
NCT03704688	Trial of Trametinib and Ponatinib in Patients With *KRAS* Mutant Advanced Non-Small Cell Lung Cancer	MEK, Multi-tyrosine Kinase	NSCLC	Trametinib: Inhibits MEK signaling downstream of *KRAS*. Ponatinib: Targets multiple tyrosine kinases and inhibits signaling
NCT03829410	Onvansertib in Combination With FOLFIRI and Bevacizumab for Second Line Treatment of Metastatic Colorectal Cancer Patients With a *KRAS* Mutation	PLK1, VEGF	CRC	Onvansertib: Selectively inhibits PLK1 causing mitotic arrest and cell death. Bevacizumab: Binds to VEGF and reduces blood supply to tumors. FOLFIRI: Chemotherapeutic
NCT04111458	A Study to Test Different Doses of BI 1701963 Alone and Combined With Trametinib in Patients With Different Types of Advanced Cancer (Solid Tumours With *KRAS* Mutation)	SOS1, MEK	NSCLC, solid tumors	BI 1701963: Inhibits *KRAS* binding to SOS1, leading to *KRAS* inactivation. Trametinib: Inhibits MEK signaling downstream of *KRAS*

## Data Availability

Not applicable.
